# Health Disparities in Alzheimer's Disease: A Wakeup Call for Transformation

**DOI:** 10.1093/geroni/igab046.1078

**Published:** 2021-12-17

**Authors:** Allison Gibson, Lenora Smith, Robert Turner

## Abstract

It is well documented in the scientific literature that health disparities exist within the Alzheimer’s disease and related dementias (ADRD) population, particularly among socially disadvantaged individuals experiencing limited opportunities to achieve optimal health. In this symposium, presenters will introduce some of the significant health disparities observed across varying ADRD research. The first presentation, by Robinson-Lane and colleagues, examines caregiving coping and health among Black ADRD families. Findings suggest in addition to traditional stress and coping strategies, additional interventions are needed that improve physical health for family caregivers. Next, Yu and colleagues will discuss the higher levels of emotional distress reported among individuals diagnosed with mild cognitive impairment, compared to their cognitively normal counterparts. In the third presentation, Lin and colleagues share their work on changes in dementia-related behavioral symptoms observed by hospice staff during COVID-19. The pandemic has affected nearly every aspect of healthcare delivery, and many hospice staff are reporting patients diagnosed with dementia have also felt the effects. Next, Xu et al identified that non-Hispanic Black older adults in their help-seeking behaviors and diagnosis process of ADRD, and often were not seen in healthcare settings by an ADRD specialist compared to their White counterparts. In the final session, Agboji will speak on the issue on findings from a systematic examination of the literature that demonstrates apathy being underrecognized and undertreated in healthcare settings. This symposium will conclude with a discussion on how researchers can disrupt these disparities by promoting health equity across ADRD healthcare and social services.

